# Eel osmotic stress transcriptional factor 1 (Ostf1) is highly expressed in gill mitochondria-rich cells, where ERK phosphorylated

**DOI:** 10.1186/1742-9994-9-3

**Published:** 2012-03-10

**Authors:** William KF Tse, Sheung C Chow, Chris KC Wong

**Affiliations:** 1 Department of Biology, Hong Kong Baptist University, Kowloon Tong, Hong Kong; 2 Center for Regenerative Medicine, Massachusetts General Hospital, Harvard Medical School, Boston, Massachusetts, USA

**Keywords:** Immunohistochemical staining, Mitochondria-rich cells, Osmotic stress transcriptional factor 1

## Abstract

**Background:**

Osmotic stress transcriptional factor 1 (Ostf1) was firstly identified in tilapia in 2005. Then numerous studies have investigated its regulation and expression profile in fish gill tissues in related to osmoregulation. Generally, hyperosmotic stress induced *ostf1 *mRNA expression level, however there is no report studying the cellular localization of Ostf1 expression in any osmoregulatory tissue. In this study immunohistochemical (IHC) approach was used to study the cellular localization of Ostf1 in gill cells of Japanese eels.

**Findings:**

Ostf1 protein was found to be localized in branchial mitochondria-rich/chloride cell (MRC/CC) as revealed by Naα5 and CFTR co-localization. The protein was detectable at day 3 after fresh water to seawater transfer and was mainly localized in MRCs. Moreover, elevated levels of extracellular signal regulated kinase (ERK) phosphorylation was observed at day 3 of the transfer and was co-localized with MRCs.

**Conclusions:**

Our data identified Ostf1 expression in gill MRCs. The observation supports the role of Ostf1 in osmosensing and/or osmoregulation in fish gills, particularly its functional relationship with MRCs. The observation of the co-expression of pERK and Ostf1 in MRCs suggests a cross-talk mechanism between the mitogen-activated protein kinases (MAPKs) and Ostf1 in response to hyperosmotic challenge. To summarize, this report has addressed the cellular localization of Ostf1 and provides evidence to illustrate the involvement of Ostf1 and ERK on osmosensing and osmoregulatory function of gill MRCs.

## Background

Osmotic stress transcriptional factor 1 (Ostf1) was discovered through subtractive hybridization to compare the gene expression profiles between gill tissues of freshwater and seawater adapted tilapia [1]. Ostf1 is then classified as an immediate early gene in response to hyperosmotic challenge in fish [2]. Our laboratory has cloned the eel Ostf1 and conducted several *in vivo *and *in vitro *experiments to unfold the regulation of the gene expression in fish gills. We have shown that *ostf1 *mRNA expression levels were increased in both gill tissues and primary gill cell culture upon hyperosmotic challenge [3,4]. In the other study, we have also shown that hyperosmotic challenge stimulated phosphorylation of extracellular signal regulated kinase (ERK) in primary eel gill cell culture [5]. The data suggest the potential importance of ERK in osmosensing and the regulation of its downstream targets. Although the importance of ERK signaling and Ostf1 expression was highlighted, currently no data on the cellular localization of Ostf1 and ERK phosphorylation in fish gills are reported.

During freshwater to seawater acclimation, the number of gill mitochondria-rich cells (MRCs) is significantly increased. The cells are believed to play an important role in hypo-osmoregulation via the expression of various ion transporters and channels to facilitate ion transport [6-8]. Using Percoll isolation and real-time RT-PCR, we reported that eel gill MRCs expressed higher level of *ostf1 *mRNA than the gill pavement cells [4]. On the basis of these observations, we hypothesized that gill MRCs utilize ERK signaling as one of the osmosensing cascade, to mediate Ostf1 and ion transporter/channel expression. By using double immunohistochemical (IHC) staining, we identified gill MRCs and examined the spatial and temporal co-localization of Ostf1 and p-ERK at different time points after freshwater to seawater transfer. In this short report, we show the co-localization of Ostf1 and pERK in gill MRCs. The observation extends our previous published data, and also provides new data on the temporal and spatial interaction of the osmotic stress signal, pERK and Ostf1 in gill MRCs.

## Result and discussion

### Ostf1 expression is localized in gill MRCs in the course of freshwater to seawater transfer

**Figure 1:**
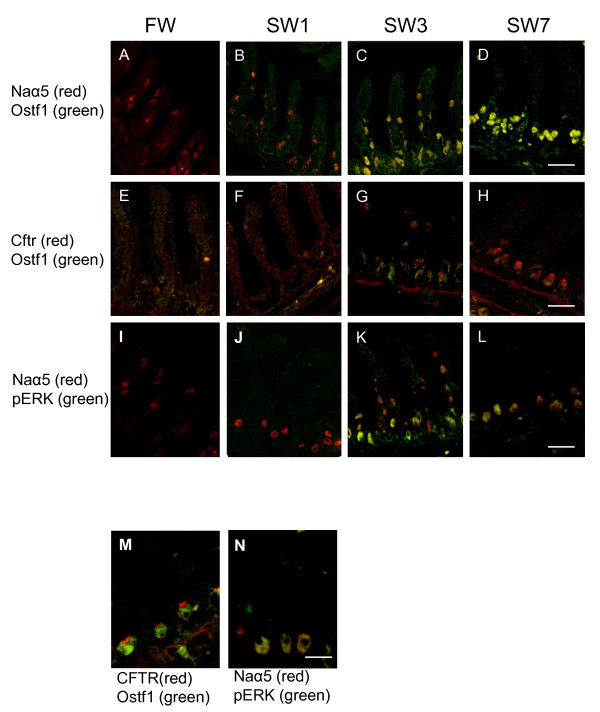
**Double immunohistochemical staining of eel gill sections, (A-D) Na^+^/K^+^-ATPase α-subunits (Naα5) and osmotic stress transcriptional factor 1 (Ostf1), (E-H) Ostf1 and cystic fibrosis transmembrane regulator (Cftr), (I-L) Naα5 and phosphorylated extracellular signal regulated kinase (p-ERK) prepared from seawater acclimating (day1, day 3, and day 7) Japanese eels (*Anguilla japonica*)**. The figures represented the general trends of four independent experiments. ***(A-H) Eel Ostf1 expressed in most cytoplasm of MRCs but not at the apical pit after day 3 freshwater to seawater transfer***. (A-D) Double staining of Naα5 and Ostf1. Red color indicated the MRCs, which were positively stained by Naα5 antibody. Increase of MRCs in primary filament region was observed after the transfer. Additionally, Ostf1 staining was marked by green color, which expressed and co-localized with MRCs (yellow) from day 3 after fresh water to seawater transfer. (E-H) Double-staining of Cftr and Ostf1. Red color marked Cftr, which started its expression at apical pit of MRCs from day 3 after fresh water to seawater transfer. The Ostf1 expressed cells were recognized by green color, which did not overlap with Cftr staining at the apical pits of MRCs. Clear red color at the apical pits was observed. ***(I-L)Induction of p-ERK expressed in gill cells after day 3 of fresh water to seawater transfer***. Double-staining of Naα5 and p-ERK. Red color marked MRCs. Green color indicated p-ERK positively stained cells, which started its expression at day 3 after fresh water to seawater transfer. It should be noted that only some of the MRCs showed ERK phosphorylation (yellow) at day 3, while all MRCs showed ERK phosphorylated at day 7 after fresh water to seawater transfer. ***(M) Magnified image of Ostf1 in MRCs*. **It clearly showed that the Ostf1 (green) did not co-localized at the apical pits with Cftr (red). ***(N) Magnified image of ERK phosphorylated MRCs***. At day 7 after fresh water to seawater transfer, p-ERK expressed in MRCs. Scale bars = 100 μm (A-D), 80 μm (E-L), and 50 μm (M-N).

To reveal the cellular localization of Ostf1 expression in fish gills, we performed IHC staining. MRCs were identified by both Naα5 and CFTR antibodies, those are the most useful tools to study MRCs in different fish species [7,8]. Together with the commercial available Ostf1 antibody, double staining with Naα5 or CFTR was conducted. In tracking the pattern of Naα5 staining in the gill sections of seawater acclimating fish, the number of the positively stained cells were reduced in the secondary lamellae but was increased in the primary filament (Figure 1A-1D). This observation is consistent with our previous report, indicating that hyperosmotic challenge activated gill cell-remodeling to facilitate salt excretion process [7]. The appearance of cystic fibrosis transmembrane regulator (CFTR)-positively stained MRCs in the primary filaments at day 3 of the seawater transfer supports this claim (Figure 1E-1H). The specific staining of CFTR was observed to be localized at the apical pits of gill MRCs. In the IHC staining, Ostf1 protein was detectable at day 3 of the seawater transfer and its expression was found to be localized in MRCs. The co-localizations of the Naα5/CFTR and Ostf1 proteins in MRCs were shown (Figure 1C-1D, 1G-1H). Furthermore our data showed that Ostf1 protein was detectable in the cytoplasm of MRCs (Figure 1E-1H), except the apical region of the cells (Figure 1M). It is not surprised that the Ostf1 staining observed in most cytoplasm of MRCs, as other ion transporters such as sodium bicarbonate co-transporter and aquaporin 3 had been reported to have similar staining pattern. It is probably due to the extensive internal membrane folding of MRCs [7,8].

### Induction of ERK phosphorylation in gill MRCs after freshwater to seawater transfer

Hyperosmotic stress triggers phosphorylation of different mitogen-activated protein kinases (MAPKs) [9,10]. *In vitro *studies of eel primary gill cell culture have shown that hypertonic challenge activated ERK phosphorylation within 2 minutes and returned back to the basal level in 30 minutes [5]. Using IHC staining in the *in vivo *whole animal experiment, the present study reported the cellular localization of phosphorylated ERK in gill tissues at day 3 after freshwater to seawater transfer (Figure 1I-1L). The onset and the duration of MAPK activation might be difference between the *in vitro *and *in vivo *studies. It can be explained by the differences in the experimental settings, in the methods of preparation and detection as well as in the time-window of cellular signal activation. In general, the experimental setting of *in vitro *primary gill cell culture provides a direct exposure of the monolayer cells to osmotic challenges, leading to a fast cellular response to the stimulation. On the contrary, in the whole animal *in vivo *studies, although gill tissue is also in direct contact with the external environment, the tissues were mostly protected by mucous secretion to minimize the magnitude of osmotic shock. In addition, the existence of other compensatory mechanisms in fish for anisosmotic adaptation might result in a lesser osmotic impact and so a relatively slower response from the gill cells. Moreover, the use of different tissue preparation and protein detection methods would lead to different extents of protein preservation and sensitivity of the detection. There shall be a great difference when comparing the data of IHC and Western blotting [7,8]. Furthermore, the detection of early ERK activation in primary gill cell culture does not mean inconsistence to the appearance of phosphorylated ERK in gill MRCs at day 3 of the seawater transfer. This can be explained by the biphasic response of ERK signaling, orchestrating diverse stimuli from osmotic challenge and gill remodeling processes. With the benefit of hindsight, the activation of ERK-signaling during gill remodeling highlights the importance of this signaling pathway in osmosensing and osmoregulatory function of the cells. It is particularly true that numerous reports have shown the importance of MAPK pathways in the regulation of various ion channels/transporters, including aquaporin, Cftr, sodium-hydrogen exchanger, and Na^+^/K^+^-ATPase [11-14]. It is noteworthy that not all MRCs showed elevated phosphorylated ERK at day 3 of the seawater transfer (Figure 1K). However, all MRCs were p-ERK positive at day 7 of the transfer (Figure 1L-1N). Nevertheless, the co-localization of Ostf1 and pERK in MRCs that we reported in this study, suggests the co-operative function of Ostf1 and pERK in MRCs to facilitate functional transformation to stimulate different ion channels and transporters for long-term adaptation.

## Materials and methods

### Animals

Japanese eels weighing 500-600 g were bought from the local fish market (Lok Fu, Hong Kong) and kept in two 40 L fiberglass tanks supplied with charcoal-filtered aerated fresh water at 18-20°C under a 12 h light: 12 h dark photoperiod for at least 3 weeks. Ten liters of the water were changed every 3 days. Some fish (n = 12) were then transferred directly to freshwater tank (as the transfer control) and seawater tanks. Both freshwater and seawater transferred fish were sampled at designated time points (days 1, 3, and 7). Three fish were samples at each time point from the freshwater and the seawater tanks. The experiment was then repeated independently for 4 times. The fish were euthanized with 0.1% MS222 in a plastic container and the gills were perfused with buffered saline (130 mM NaCl, 2.5 mM KCl, 5 mM NaHCO3, 2.5 mM glucose, 2 mM EDTA, and 10 mM Hepes, pH 7.7) to remove blood cells. Gill arches were excised and washed. One of the gill arches (first left gill arch) was fixed for IHC staining. All experimental procedures on the fish were approved by Hong Kong Baptist University, Hong Kong Special Administrative Region.

### Immunohistochemical (IHC) staining

A paraformaldehyde-fixed wax section of the gill tissue (6 μm thickness) was de-waxed, rehydrated in graded ethanol, and rinsed in phosphate-buffered saline (PBS). The staining procedure involved pretreatment of the tissue section with 10% normal goat serum in PBS to reduce nonspecific binding, followed by an hour incubation at room temperature with antiserum (i.e. mouse anti-Na^+^/K^+^-ATPase α-subunits (Naα5) (1:100, Hybridoma Bank), rabbit anti-pERK (1:100, Cell Signaling), and rabbit anti-GilZ/TilZ (1:100, Abcam). The section was rinsed in PBS, incubated with Alexa Fluor 488 or 568 goat anti-mouse/anti-rabbit IgG (1:200, Molecular Probes), and examined by a laser confocal microscope (Fluoview, Olympics). The slides were washed 3 times for 15 minutes each in PBS after each antiserum application. The control procedure included the application of non-immune mouse or rabbit serum (Sigma) (Additional file 1: Figure S1). Eel Ostf1 encodes TSC22 (GilZ/TilZ) domain, which was recognized by the anti-GilZ/TilZ antibody. The antibody recognizes the carboxyl terminus of mouse GilZ/TilZ that shares high amino acid sequences homology in eel GIlZ/TilZ domain. The specificity of the antibody was confirmed by Western blotting. Specific band at 30 kDa was marked (Additional file 1: Figure S2).

## Abbreviations

CC: Chloride cell; ERK: Extracellular signal regulated kinase; IHC: Immunohistochemical; MAPK: Mitogen-activated protein kinase; MRC: Mitochondria-rich cell; Ostf1: Osmotic stress transcriptional factor 1.

## Competing interests

The authors declare that they have no competing interests.

## Authors' contributions

CKCW and WKFT designed the experiment and wrote the manuscript. WKFT carried out the IHC experiments. SCC preformed the Western blotting. All authors read and approved the final manuscript.

## Supplementary Material

Additional file 1 **Figure S1 Negative control of immunohistochemical staining experiments using the non-immune mouse or rabbit serum**. No positive signals of Ostf1, pERK, and Cftr were found in the gill epithelia prepared from Japanese eels (Anguilla japonica) acclimated in seawater (day 7). Scale bar = 80 μm. Figure S2: The specificity of the Ostf1 antibody was confirmed by Western blotting. A band at 30 kDa was identified in the gill sample. Actin was used as the loading control. Click here for file
