# Supplementary figures and images for: Eel osmotic stress transcriptional factor 1 (Ostf1) is highly expressed in gill mitochondria-rich cells, where ERK phosphorylated
Source: Front Zool. 2012 Mar 10;9:3. doi: 10.1186/1742-9994-9-3 (PMC3315740; doi:10.1186/1742-9994-9-3)

**Supplementary Figure 1**

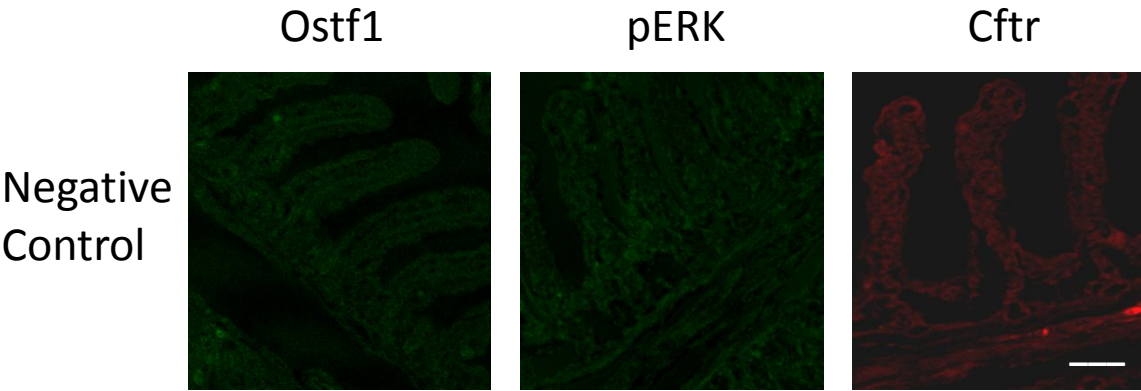

**Supplementary Figure 2**

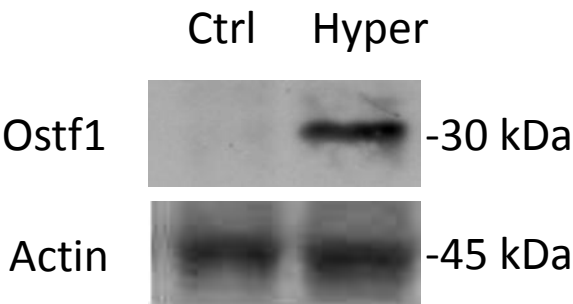

Supplement: Additional file 1 — Figure S1 Negative control of immunohistochemical staining experiments using the non-immune mouse or rabbit serum. No positive signals of Ostf1, pERK, and Cftr were found in the gill epithelia prepared from Japanese eels (Anguilla japonica) acclimated in seawater (day 7). Scale bar = 80 μm. Figure S2: The specificity of the Ostf1 antibody was confirmed by Western blotting. A band at 30 kDa was identified in the gill sample. Actin was used as the loading control. [file 1742-9994-9-3-S1.PDF]
